# From Pre-Foaling to Late Pregnancy: Cortisol, DHEA(S), Progesterone, 17-β-Estradiol, and Allopregnanolone Hair Concentration Profiles in Standardbred Mares

**DOI:** 10.3390/ani15030324

**Published:** 2025-01-23

**Authors:** Maria Cristina Veronesi, Alessio Cotticelli, Isabella Pividori, Matilde Giombolini, Mirco Corazzin, Letizia Ellero, Tanja Peric

**Affiliations:** 1 Department of Veterinary Medicine and Animal Sciences, Università degli Studi di Milano, 26900 Lodi, Italy; maria.veronesi@unimi.it; 2 Department of Veterinary Medicine and Animal Production, Federico II University, 80137 Naples, Italy; alessio.cotticelli@unina.it; 3 Department of Agricultural, Food, Environmental and Animal Sciences, University of Udine, 33100 Udine, Italy; matilde.giombolini@uniud.it (M.G.); mirco.corazzin@uniud.it (M.C.); letizia.ellero@uniud.it (L.E.); tanja.peric@uniud.it (T.P.)

**Keywords:** hair, horse, late pregnancy, cortisol, DHEA(S), progesterone, 17-β-estradiol, allopregnanolone

## Abstract

Pregnancy and parturition represent two important physiologic phases in female mammals, in which metabolic, behavioral, and endocrinologic changes should occur in perfect timing and interaction, leading to a normal course of gestation and the occurrence of parturition at term, allowing the birth of mature and viable offspring. The present study aimed to describe the hormonal changes recorded in the hair occurring in mares from pre-foaling to late pregnancy. The hair cortisol (C) concentrations did not show any significant variations throughout the study, while dehydroepiandrosterone (sulfate) (DHEA(S)) hair concentrations showed an increase from ST-1 to ST4 (*p* < 0.01) followed by a significant decrease at ST6 (*p* < 0.01). From ST7 to ST8, hair DHEA(S) concentrations increased significantly (*p* < 0.01). In ST7 the cortisol/DHEA(S) ratio*100 was higher than in the other sampling times (*p* < 0.01) except for ST0, in which the ratio was similar to ST7. Hair progesterone (P4), 17-β-estradiol (E2), and allopregnanolone (AlloP) concentrations showed similar patterns through time with the exception of some differences between them at the end of the sampling period. The results showed that hormones fluctuations in the hair mimicked those observed previously in plasma making hair interesting for retrospective studies both on mares and foals during gestation without invasiveness and adding a complementary tool in studying the feto-maternal relationship.

## Simple Summary

Most of the studies reporting hormonal changes that occur in a pregnant mare have been performed on blood. Considering the advantage of hair as a non-invasive, retrospective, and long timeframe matrix, the present study aimed to describe concentrations of important hormones for the equine gestation and parturition in hair. The results confirmed hair as a reliable matrix for longitudinal investigations of the past accumulated hormones without causing harm to the animals and with minimal disturbance. Moreover, the fluctuations of cortisol, dehydroepiandrosterone (sulfate), progesterone, 17-β-estradiol, and allopregnanolone concentrations in hair mimicked those already described in previous studies on plasma, but new interesting questions about the physiologic hormonal changes during the equine pregnancy arose and deserve further investigation.

## 1. Introduction

Pregnancy and parturition represent two important physiologic phases in female mammals, in which metabolic, behavioral, and endocrinologic changes should occur in perfect timing and interaction, leading to a normal course of gestation and the occurrence of parturition at term, allowing the birth of mature and viable offspring. Because of the mare’s reproductive biology, a new pregnancy usually starts early after parturition, so the puerperium coincides not only with lactation and nursing foal but also with the new pregnancy. The time from pre-foaling to the subsequent pregnancy establishment represents, therefore, a very interesting period for the study of hormonal changes.

Traditionally, endocrine investigations have been performed on blood, with the advantage of detecting acute changes, such as changes occurring during the process of parturition. On the other hand, blood plasma does not represent the best option for the longitudinal studies of long physiologic conditions such as pregnancy. On one hand, in fact, the hormonal analysis performed on blood provides information about that single punctual sampling time that does not mirror changes occurring slowly in a longer timeframe. On the other hand, the necessary repetition of multiple blood sampling is an issue from the animal welfare standpoint, particularly important during gestation and during maternity, as reported in dogs [1]. This aspect can be overcome with the use of the hair collected every 30 days [1]. In response to this need, the hair proved to be a reliable matrix for longitudinal studies investigating long-term hormonal changes, not influenced by acute or circadian rhythms. Hair sampling is non-invasive and painless compared to traditional blood sample collection; the procedure is simple, and samples can be stored at room temperature until analysis. Hair analysis represents a consolidated methodological approach for the non-invasive measurement of steroids (and alternatively to on-ground collected feces), allowing for a retrospective analysis of the total exposure to steroids over time and avoiding of the influence of acute events or circadian fluctuations [2]. As a cumulative matrix, hair incorporates hormones and other circulating substances during its growth period [3,4,5] even though the precise mechanisms by which lipophilic steroid hormones are incorporated into hair are still not fully understood.

In recent decades, many studies have focused especially on the measurement of hair cortisol (C), the main glucocorticoid in mammals [6,7], commonly used as a physiological indicator of stress. When homeostasis is threatened by the actions of various external or internal stressors, cortisol is secreted in response to the activation of the hypothalamic–pituitary–adrenal (HPA) axis [8,9]. However, apart from being the endpoint of stress, cortisol plays an important role in many physiological processes, among them the preparation for parturition and birth [10]. At the end of pregnancy, in response to the ACTH stimulation, the mature fetal adrenal glands start to produce cortisol, which will be responsible for a sudden decrease in progestins in the maternal circulation as the concentration of cortisol increases [11,12]. Other important steroids during equine gestation and parturition are progesterone (P4) and 17β-estradiol (E2) [13,14]. Other than that, allopregnanolone (AlloP) [15], a P4 metabolite [16], was also described as a hormone strictly connected with mare gestation [17], while the maternal circulating concentrations of dehydroepiandrosterone (DHEA) and its sulfated form, DHEA-sulfate (DHEA-S), are the result of fetal gonad production [18].

Given that hair incorporates systemic steroids into the growing hair shaft from blood vessels via passive diffusion during its growing phase—anagen [4]—our hypothesis is that it is possible to study the variations in some of the most important hormones of gestation and foaling in the hair as it has been already performed previously but with very invasive sampling. Thus, the present study aimed to describe for the first time the hair concentrations profile of cortisol, DHEA(S) (considered as the sum of DHEA and DHEA-S), progesterone, allopregnanolone, and 17-β-estradiol from pre-foaling to late pregnancy in mares as a retrospective measure of the hypothalamic–pituitary–adrenal and hypothalamic–pituitary–gonadal axes activity. Moreover, because of the recognized role of the cortisol/DHEA(S) ratio as one new potential biomarker of resilience and allostatic load [7], this parameter was also considered.

## 2. Materials and Methods

Although hair sampling is a non-invasive and non-troublesome procedure, the study has been carried out following the EU Directive 2010/63/EU and the Italian legislation on animal care (Legislative Decree No. 26 of 4 March 2014).

### 2.1. Animals

This study was performed on 11 Italian Standardbred mares, 7–10 years old, from a single stable located in Northern Italy, during spring 2023. Only multiparous, late pregnant mares with the normal course of pregnancy, normal gestation length, and normal spontaneous delivery of viable foals occurring in March–April were included in the study. All foalings occurred under staff surveillance enabling the record of parameters related to parturition and the newborn. The mares and the foals were housed in single boxes during the first 5 days after foaling and then transferred into common paddocks in which mares and foals were grouped according to the age of the foals. Both mares and foals were checked daily for health conditions and normal development. Mares had access to pasture, and they were fed hay ad libitum throughout pregnancy; from the last three months of pregnancy until the fourth postpartum month a commercial for broodmares’ fodder was added daily (4 kg/mare).

Water was available ad libitum. Mares were mated at the first or the second heat postpartum period, and all became pregnant within 2 months of the postpartum period.

At 6 months of age, foals and mares were abruptly separated, and foals were moved to a different stable, with mares out of earshot of the foal and foals stabled in single boxes with free access to outdoor paddocks.

### 2.2. Hair Sample Collection

Individual hair samples were collected about 30 days before the scheduled delivery date (ST-1) from the wither’s region, shaving the hair close to the skin with an electric razor. The second sampling was performed at foaling (ST0) from the same area to obtain only re-grown hair. Re-grown hair samples were subsequently collected every 30 days from ST1 to ST8. All samples were stored in a dark environment, in paper envelopes, at room temperature until the end of the study, when they were analyzed.

### 2.3. Hair Washing Procedure and Extraction

Hair samples were washed in 3 mL isopropanol (Merck KGaA, Darmstadt, Germany) to ensure the removal of any steroids on their surface while the steroids from hair were extracted with methanol. In brief, 20 mg of trimmed hair was placed in a glass vial along with 3 mL of methanol (Merck KGaA, Darmstadt, Germany). The vials were incubated at 37 °C for 16 h. Next, the liquid in the vial was evaporated to dryness at 37 °C under an airstream suction hood. The remaining residue was dissolved in 0.6 mL of 0.05 M phosphate-buffered saline (PBS) at pH 7.5.

### 2.4. Hormone Analyses

The concentrations of cortisol [19], DHEA(S), P4, and 17-β-estradiol were measured using a solid-phase microtiter RIA. In brief, a 96-well microtiter plate (OptiPlate; PerkinElmer Life Sciences Inc., Waltham, MA, USA) was coated with goat anti-rabbit γ-globulin serum diluted 1:1000 in 0.15 mM sodium acetate buffer (pH 9), and the plate was incubated overnight at 4 °C. The plate was then washed twice with RIA buffer (pH 7.5) and incubated overnight at 4 °C with 200 μL of the antibody serum diluted 1:20,000 for cortisol, 1:80,000 for DHEA(S), 1:8000 for P4, and 1:80,000 for 17-β-estradiol. The rabbit anti-cortisol antibody used was obtained from Biogenesis Ltd. (Poole, UK). The cross-reactivity of the anti-cortisol antibody with other steroids was as follows: cortisol, 100%; corticosterone, 1.8%; and aldosterone, <0.02%. The rabbit anti-DHEA(S), anti-P4, and anti-17-β-estradiol antibodies used were obtained from Analytical Antibodies (Bologna, Italy). The cross-reactivities of the anti-DHEA(S) antibody with steroids were as follows: DHEA-S, 100%; DHEA, 100%; DHEA 3-glucuronide, 15%; androstenedione, 5.9%; pregnenolone, 0.3%; epiandrosterone 3-glucuronide, 2.7%; androsterone sulfate, 2.9%; cortisone, <0.001%; cholesterol, 0.00001%; and cholesterol oleate, 0.00001%. Regarding the analysis of DHEA(S), because the antibody showed a noticeable cross-reactivity for both DHEA and DHEA-S, the hormonal concentrations reported in this study have to be regarded as immunoreactive “DHEA(S)”. In addition to that for progesterone, the anti-P4 antibody shows the following cross-reactivity with other progestogens that are known to be found in the pregnant mare [17]: 11 α-OH-progesterone, 87%; 11 β-OH-progesterone 46%; 17 α-OH-progesterone, 0.4%; 5 α-OH-progesterone, <1%; 20 α-OH-progesterone, 0.04%; other progestogens <0.1%; testosterone, 0.08%; cortisol, <0.01%; estradiol-17-β, <0.01%; estradiol-17-α, <0.01%; and estrone, <0.01%. The antiserum was specific for 17-β-estradiol and had the following cross-reactivity: 17-α-estradiol, 0.62%; estrone, 1.5%; estrone-3-sulphate, 0.3%; estriol, 0.8%; estriol-3-sulphate. 0.03%. After having washed the plate with RIA buffer, the standards (5–200 pg/well), the quality control extract, the test extracts, and the tracer (hydrocortisone {cortisol [1,2,6,7-^3^H (N)]-}, DHEA-S [1,2,6,7-^3^H (N)], progesterone [1,2,6,7-^3^H (N)], and 17-β-estradiol [2,4,6,7,16,17-^3^H (N)]; PerkinElmer Life Sciences Inc., Waltham, MA, USA) were added, and the plate was incubated overnight at 4 °C. The bound hormone was separated from the free hormone by decanting and washing the wells in RIA buffer. After the addition of 200 μL of scintillation cocktail, the plate was counted on a β-counter (Top-Count; PerkinElmer Life Sciences Inc., Waltham, MA, USA). The intra- and inter-assay coefficients of variation were 3.6 and 9.8%, 3.2 and 11.8%, 3.4 and 8.2%, and 3.8 and 9.7% for cortisol, DHEA(S), P4, and 17-β-estradiol, respectively. The sensitivity of the assay was 24.6 pg/mL, 10.8 pg/mL, 4.6 pg/mL, and 14.7 pg/mL for cortisol, DHEA(S), P4, and 17-β-estradiol, respectively. The relationship between hair cortisol, hair DHEA(S), hair P4, hair 17-β-estradiol, and the respective standard curves determined through linear regression were linear, with a correlation coefficient of r = 0.99. The model was described by the equation y = 1.00x + 4.14, y = 0.99x + 0.28, y = 0.98x + 0.36, y = 0.98x − 0.94 for cortisol, DHEA(S), P4, and 17-β-estradiol, respectively.

Allopregnanolone concentrations were evaluated by the Enzyme Immunoassay method using a commercial kit DetectX^®^ from Arbor Assays Inc (Product number: K061-H5, Ann Arbor, MI, USA) following the manufacturer’s instructions and as validated for horse hair by our lab [20]. The assay sensitivity was 50.4 pg/mL. The intra- and inter-assay coefficients of variation were 6.4% and 11.0%, respectively.

### 2.5. Statistical Analysis

Statistical analysis was performed using SPSS (28.0) for Windows 10 (SPSS Inc., Chicago, IL, USA). The normality of data distribution and homogeneity of variance were tested using the Shapiro–Wilk and Levene tests, respectively. When necessary, data were transformed for parametric testing. The possible changes in hair hormones concentrations were analyzed using a linear mixed model considering the sampling time (ST-1, ST0, ST1, ST2, ST3, ST4, ST5, ST6, ST7, and ST8) as repeated measures. The post hoc multiple comparisons were performed using the Ryan-Holm–Sidak procedure as suggested by Atkinson (2002) [21] and Ludbrook (1998) [22]. Significance was set for *p* < 0.05.

## 3. Results

**Figure 1:**
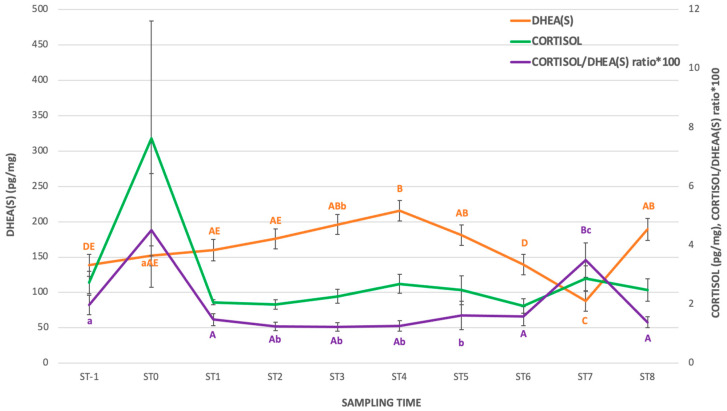
Hair concentrations (mean ± SE) of C (cortisol) and DHEA(S) and the cortisol/DHEA(S) ratio*100 from pre-foaling to late pregnancy in the 11 mares enrolled in the study. ^a, b, c^ denotes significant differences for *p* < 0.05; ^A, B, C, D, E^ denotes significant differences for *p* < 0.01.

**Figure 2:**
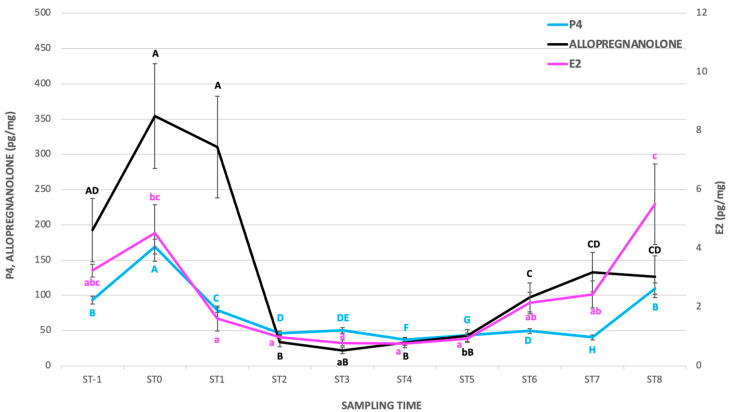
Hair concentrations (mean ± SE) of progesterone (P4), 17-β-estradiol (E2), and allopregnanolone (AlloP) from pre-foaling to late pregnancy in the 11 mares enrolled in the study. ^a, b, c,^ denotes significant differences for *p* < 0.05; ^A, B, C, D, E, F, G, H^ denotes significant differences for *p* < 0.01.

Data about the profiles (mean ± SE) of C and DHEA(S) hair concentrations and the cortisol/DHEA(S) ratio*100 are reported in Figure 1, while the profiles of P4, E2, and AlloP hair concentrations are reported in Figure 2.

As shown in Figure 1, the hair C concentrations did not show any significant variations throughout the study, while DHEA(S) hair concentrations showed an increase from ST-1 to ST4 (*p* < 0.01) followed by a significant decrease at ST6 (*p* < 0.01). From ST7 to ST8, hair DHEA(S) concentrations increased significantly (*p* < 0.01). In ST7, the cortisol/DHEA(S) ratio*100 was higher than in the other sampling times (*p* < 0.01), except for ST0, in which the ratio was similar to ST7.

The highest hair P4 concentrations were those recorded at ST0. A rise was observed at the end of the experiment; in particular, P4 concentrations at ST8 were higher than that observed at ST7. Higher hair E2 concentrations were observed at ST0 in comparison to ST1-ST5 (*p* < 0.05). Conversely, the E2 concentrations at ST8 reached similar values to those observed at ST-1 and ST0). The hair AlloP concentrations recorded at ST-1, ST0, and ST1 were higher than those observed at ST2, ST3, ST4 and ST5. Afterward, the values recorded at ST7 and ST8 were similar to the values at ST-1.

## 4. Discussion

Although the use of hair to assess cortisol concentrations has received great interest in humans, domestic, and wild mammals [4,23], its use for the analysis of sexual hormones and other steroids received little attention. The use of the hair as a matrix of hormone measurement is particularly interesting for studies about physiologic conditions such as the time around foaling and during pregnancy, in which the disturbance to the animals must be limited as much as possible. However, to the best of the authors’ knowledge, this is the first study that reports the pre- and post-foaling changes in C, DHEA(S), P4, AlloP, and E2 in the hair of mares.

Although plasma C concentrations have been reported to increase in mares at parturition [12], in the present study, the hair C concentrations did not show any variation over time. This could be related to the characteristic of the hair as a biological matrix that, as described previously, is not subjected to acute changes in the systemic hormone concentration. Specifically, Nagel et al. (2012) [12] reported that plasma cortisol concentrations rose only 4 days before delivery with a sharp decrease already within 24 h after foaling. These short-timed changes could have been masked in the hair, which provides the picture of concentrations accumulated in the time frame of timing collection (i.e., 30 days). Values observed at ST0 coincide with those described in mares by Lanci et al. (2022) [24] in hair collected at parturition. In the present study, the postpartum period coincides with maternity, lactation, and the beginning of a new pregnancy from ST1–ST2. The absence of significant changes in hair C concentrations along the time of study seems to suggest that, in these mares, none of these physiologic events typical of the studied period activated the HPA axis. Interestingly, the C concentrations measured at ST7, which refers to C concentrations accumulated in the hair from 6 and 7 months of the postpartum period, did not show significant increases that could have been related to the separation of foals from the mothers at weaning, suggesting the absence of chronic HPA axis activation. Previous studies performed using plasma cortisol concentration measurements also showed low cortisol concentrations during the postpartum period [25,26]. Other studies focused on the timing of mother-to-foal separation at weaning, demonstrating the absence of negative effects on both mothers and foals when weaning is practiced gradually [27,28,29]. In the present study, in which weaning was not gradual but abrupt, no significant increases in cortisol have been detected. This finding could be explained by the retrospective long-term accumulation (30 days) of the hormone in the hair, or even by the fact that all the mares were multiparous and, therefore, could have been familiar with the event of weaning. This aspect deserves interest and could be intriguing to verify whether the same results could have been drawn from primiparous mares.

Interestingly, the DHEA(S) hair concentrations increased progressively from ST-1 to ST4. The increasing concentration of hair DHEA(S) could be related to DHEA(S) production by the fetal gonads. The fetal DHEA(S) has been reported to be aromatized in the placenta and released as estrogens in the maternal circulation [30,31]; however, some authors [18,32] suggested that a considerable amount of DHEA(S) escapes the placenta, increasing its concentrations in the maternal circulation in early pregnancy, peaking in mid-pregnancy, and then decreasing toward the end of pregnancy with the regression of the fetal gonads during late gestation [11]. Likely, a decrease in hair DHEA(S) concentrations has been observed in the present study from ST4 to ST6 after, surprisingly, a new increase similar to concentrations registered between ST1 and ST5 was detected at the last sampling at ST8.

Apart from that in ST0, the cortisol/DHEA(S) ratio showed a significant increase only at ST7 as a result of constant hair cortisol concentrations and the lowest concentration of DHEA(S). To the author’s knowledge, the increase in DHEA concentrations in the last third of the mare’s gestation is not reported, even if in humans it was reported to play a role in the initiation and acceleration of labor, together with cortisol [33]. Therefore, the findings about hair DHEA(S) and the related cortisol/DHEA(S) ratio deserve interest for future investigations.

The mare’s hair P4 concentrations observed in the present study showed the highest value at ST0, maybe reflecting the rise in plasma of metabolites of pregnenolone (P5) and P4, collectively known as progestogens [11,30], occurring in the last 20–30 days of gestation. This increase is believed to be necessary to ensure the quiescence of the equine uterus during late gestation, strongly reduced over the last few days before foaling [12,32]. During the postpartum period, the hair P4 concentrations remained low and did not show any significant increase until the last sampling time. Because during the first 5 months of equine pregnancy P4 is secreted by the primary and secondary corpora lutea [34], it could be possible to hypothesize that the circulating P4 concentrations probably do not contribute to the substantial increase in the hair P4 concentrations. On the contrary, the significant increase in the hair P4 concentrations observed at ST8 agrees with the reported increase in circulating progestogens in the last third of equine gestation [11]. The increase observed at ST8 could have also contributed to the feto-placental unit activity, which converts pregnenolone (P5) derived from the fetal circulation, in other progestogens, involved in pregnancy maintenance [11,35,36].

After an early validation study about the AlloP hair concentrations in pregnant vs. non-pregnant mares [20], to the author’s knowledge, this is the first study reporting data about the possible changes from pre-foaling to late pregnancy AlloP concentrations in the hair. The hair AlloP showed higher concentrations between ST-1 and ST1, followed by a sharp decrease from ST2 to ST5, with a subsequent new increase at ST6 with concentrations remaining stable until ST8, probably due to the activity of the feto-placental unit in the second half of pregnancy in converting P5 derived from the fetus not only to P4 but also to allopregnanolone [11]. In fact, allopregnanolone was reported as one of the major progestogens taking part in the hormonal set of the pregnant mare [17], with concentrations increasing in late gestation [14,31,32,33,37,38,39]. It should be underlined that, in the present study, all the studied mares were already pregnant from the second month after foaling; therefore, at ST6–ST8 all the mares were at 4–6 months of pregnancy.

Besides progestogens, hair E2 also showed higher concentrations at ST0, with a significant fall soon after (ST1–ST5), followed by a trend of increase, with significantly higher E2 concentrations detected at ST8 in comparison to ST1–ST5. Both the decrease after birth and the increase detected at ST8 agree with Fowden et al. (2008) [11] describing the highest estrogens circulating concentrations in the last 3 months of pregnancy with a fall immediately after birth.

The animals were selected according to strict criteria to provide a picture of changes occurring under normal conditions, reducing potential influencing hormones [5,23,40]. A potential study limit could be that mares became pregnant at the first or second heat with an influence on hormonal patterns, but given the low variability of hormone concentrations at each sampling point in the postpartum period we can conclude that there was no real influence.

## 5. Conclusions

In conclusion, the results of the present study confirmed the hair as a reliable matrix for the longitudinal investigation of retrospective accumulation of hormones, without invasiveness and with minimal disturbance for the animals. Considering the retrospective information provided by the hair, hormone fluctuations mimicked those observed in plasma, but further research is needed to more deeply understand the physiologic hormonal changes during the equine pregnancy that arose, deserving further investigations.

## Data Availability

The data presented in this study are available on reasonable request from the corresponding author.
